# Cryostorage of Mesenchymal Stem Cells and Biomedical Cell-Based Products

**DOI:** 10.3390/cells11172691

**Published:** 2022-08-29

**Authors:** Daria D. Linkova, Yulia P. Rubtsova, Marfa N. Egorikhina

**Affiliations:** Federal State Budgetary Educational Institution of Higher Education, Privolzhsky Research Medical University of the Ministry of Health of the Russian Federation, 603005 Nizhny Novgorod, Russia

**Keywords:** mesenchymal stem cells, cryoprotector, protocols for cryopreservation, tissue, scaffold

## Abstract

Mesenchymal stem cells (MSCs) manifest vast opportunities for clinical use due both to their ability for self-renewal and for effecting paracrine therapeutic benefits. At the same time, difficulties with non-recurrent generation of large numbers of cells due to the necessity for long-term MSC expansion ex vivo, or the requirement for repeated sampling of biological material from a patient significantly limits the current use of MSCs in clinical practice. One solution to these problems entails the creation of a biobank using cell cryopreservation technology. This review is aimed at analyzing and classifying literature data related to the development of protocols for the cryopreservation of various types of MSCs and tissue-engineered structures. The materials in the review show that the existing techniques and protocols for MSC cryopreservation are very diverse, which significantly complicates standardization of the entire process. Here, the selection of cryoprotectors and of cryoprotective media shows the greatest variability. Currently, it is the cryopreservation of cell suspensions that has been studied most extensively, whereas there are very few studies in the literature on the freezing of intact tissues or of tissue-engineered structures. However, even now it is possible to develop general recommendations to optimize the cryopreservation process, making it less traumatic for cells.

## 1. Introduction

Methods based on the potential of stem cells to stimulate reparative mechanisms and to restore the functions of damaged tissues or organs are widespread in regenerative medicine [1]. Multipotent mesenchymal/stromal stem cells (MSCs) are the types of stem cell most in demand; these are non-hematopoietic stem cells of an adult organism, capable of differentiating into mesodermal lines (osteocytes, adipocytes, chondrocytes), as well as into ectodermal (neurocyte) and endodermal (hepatocyte) lines [2].

MSCs manifest vast opportunities for clinical use not only due to their ability for self-renewal in vitro and their multipotent differentiation, but also, importantly, due to their specific properties. For instance, depending on the level of stimulation by neighboring cells, cytokines, or soluble factors, MSCs can have a pro- or an anti-inflammatory effect on their microenvironment. MSCs are sources of active biomolecules such as growth factors, cytokines, and chemokines, revealing their autocrine and paracrine activity [3,4,5]. Moreover, due to their production of immunoregulatory molecules, MSCs can cultivate resistance to immune cells, thus facilitating reduction in the risk of graft-versus-host disease and, as a result, effective graft retention [6,7]. All the above provide for the successful use of MSCs in many areas of medicine: in the treatment of non-healing wounds [8], autoimmune and neurodegenerative diseases [9], in osteochondral [10] and cardiovascular diseases [11], as well as in diabetes [12]. Furthermore, there are ongoing clinical trials on the use of MSCs for add-on therapies in COVID-19 treatment [13,14].

At present, there is no doubt that the microenvironment has a significant impact on the activity and functioning of MSCs. At the same time, MSCs can maintain tissue homeostasis by changing the composition and structure of the extracellular matrix. Thus, the mutual influence of the stem cells and their microenvironment constitute the “niche” concept [15,16]. This concept has become fundamental for the development of various bioengineered structures—scaffolds, which can simulate the extracellular matrix and act as artificial niches for stem cell culture [17,18,19]. The scaffold provides an environment for the cells’ three-dimensional existence, one which is much more similar to the conditions within body tissues than is flat cultivation. Such simulation of natural conditions for MSCs provides for prolongation of the life of the cells in the implantation area, as well as for an increase in the efficacy of their differentiation, secretory activity, etc. [20,21]. Thus, tissue-engineered products resulting from the use of scaffold techniques can have a significantly better regenerative potential than classical cell-based products.

At the same time, despite all the advantages of MSCs and MSC-based tissue-engineered products, a variety of difficulties occur in their clinical application. For instance, the therapeutic dose used in regenerative therapy can vary widely, for example, from 50 to 400 million stem cells [22,23]. In fact, it is impossible to extract such a large number of cells from one-donor tissue. Thus, there is a need for MSC expansion ex vivo, which can be a lengthy process [24]. This largely limits the use of MSCs in cases when a prepared graft is urgently required. Moreover, when re-introduction of MSCs is required, re-sampling of tissues from the donor may be impossible or highly problematic. Even a procedure such as liposuction, which is considered minimally invasive, can lead to severe complications, let alone those that could result from complex manipulations, such as bone marrow aspiration [25].

These problems can be solved by the creation of a bank of ready-to-use biological material. Such biobanking of MSCs provides for minimizing the preparation time of a therapeutic product, making it immediately available to patients, as well as for quality control and for the standardization of cell-based products [24]. Moreover, successful long-term storage facilitates the commercialization of therapeutic products [26]. Thus, the establishment of a cell-based material biobank will significantly empower research centers and manufacturers, as well as medical clinics as end users [27]. Biobanking typically implements technologies based on the use of low temperatures, with cryopreservation being the most widespread among them. Cryopreservation is the process of preserving biological samples by cooling them to very low temperatures [28]. This is related to the ability of cells to enter metabolic stasis at temperatures below −120 °C [29]. Here, the success of cryopreservation depends on numerous factors such as the freezing rate, the composition and concentration of the cryoprotective agents (CPAs), the temperature regime and the duration of cryopreservation, thawing, etc. This wide range of parameters has contributed to the development of many different protocols for the preservation of cell-based products.

At the same time, while many of the methods and protocols that have been developed promote successful cryopreservation of cell suspensions, there are very few studies in the literature related to the freezing of tissue-engineered structures. Such structures are complex, hence assessment of cryopreservation success for them is very difficult and differs greatly from assessment for the cells alone [30]. However, comparison and optimization of the cryopreservation protocols for various types of cell-based and tissue-engineered products can assist in the development of tissue-engineered structures with cryostable characteristics [31].

The purpose of this review is to analyze and classify the literature data related to the development of cryopreservation protocols for various types of MSCs and tissue-engineered structures, as well as to compare the efficacy of the applied biobanking protocols and temperature regimes.

## 2. Cryoprotectors and the Cryoprotecting Environment

A number of negative physical effects occur during freezing that can cause cell death and reduce the cell-based product quality. First, is the problem of dehydration and intracellular icing. Cell dehydration occurs when the freezing rate is low, and is a consequence of the water freezing out in the external environment, resulting in an increase in the concentration of dissolved substances outside the cells. High concentrations of salts in the extracellular environment lead to an osmotic pressure gradient on the cell plasma membrane; water moves out from the cells into the extracellular environment, resulting in cell dehydration [32]. By contrast, the phenomenon of intracellular water crystallization is characteristic of high freezing rates and causes an increase in the internal volume of membrane structures (lysosomes, endoplasmic reticulum, Golgi apparatus, etc.), which results in their destruction. The use of cryoprotectors, which protect cells during freezing, minimizes the adverse effects of the increased concentration of dissolved substances outside the cells and the formation of intracellular ice crystals [33].

Cryoprotectors are substances that are capable of preventing biological objects from suffering freezing damage, as well as ensuring their viability upon thawing [34].

**Figure 1:**
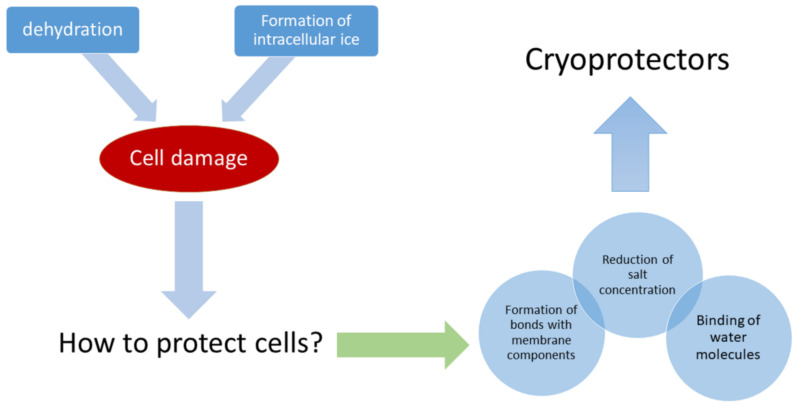
General principles of cryoprotector operation.

The general principle of a cryoprotector’s protective action on freezing is based on the following capacities (Figure 1):

- To create strong bonds with water molecules both outside and inside the cell, with these bonds being stronger than the bonds between water molecules;
- To decrease salt concentrations, thus minimizing the risk of damaging the cells’ protein structures;
- To bond with the structural components of the membrane, protecting them from being destructed by ice crystals [35].

At present, there are more than 120 substances pertaining to different classes of chemical compounds that have been identified and tested as promising cryoprotectors. These include examples of alcohols (ethylene glycol, α-propylene glycol, glycerin), amides (dimethylacetamide, urea), oxides (dimethyl sulfoxide), carbohydrates (glucose, sucrose), synthetic polymers (polyvinylpyrrolidone, ethoxylated starch, polyethylene glycol, polyethylene oxide), inorganic salts (sodium chloride, potassium chloride, calcium chloride, sodium citrate, trisodium phosphate, EDTA disodium salt) [36].

### 2.1. Classification of Cryoprotectors

**Figure 2:**
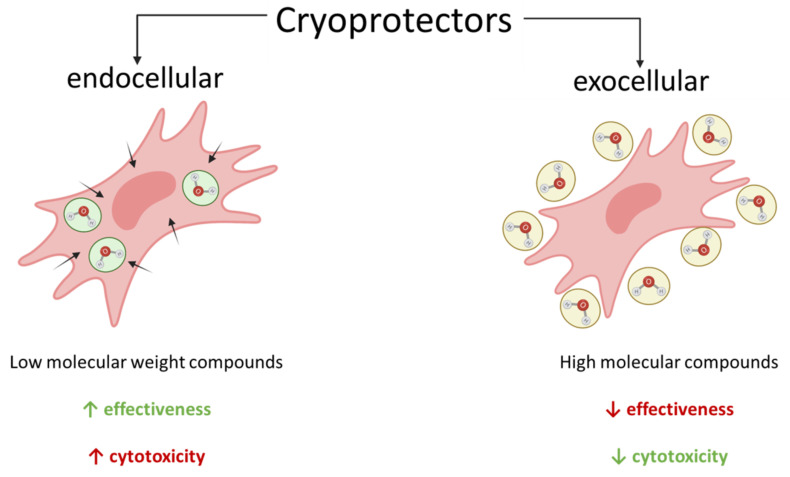
Types of cryoprotectors.

All cryoprotectors can be divided into two categories by their mode of action: (1) endocellular (penetrating) cryoprotectors, with low molecular weight, that penetrate through the cell membrane; (2) exocellular (non-penetrating) cryoprotectors, with high molecular weights, that do not penetrate through the cell membrane [37] (Figure 2).

Endocellular cryoprotectors are substances that penetrate deeply into the cell and prevent ice crystal buildup by forming hydrogen bonds with intracellular water molecules. These cryoprotectors are considered the more effective type; however, due to their high penetration capacity, they are also more toxic. The most common endocellular cryoprotectors are dimethyl sulfoxide (DMSO), glycerol, ethylene glycol, propylene glycol, 1,2-propanediol [38] (Table 1).

Exocellular cryoprotectors are macromolecular compounds that are incapable of going through the cell membrane into the cell. Their modes of action are based on the binding of extracellular water, protecting the cell from osmotic changes, inhibiting the growth of extracellular ice crystals, cell enveloping and preventing the impact of the existing crystals on it. There are two groups of substances related to exocellular cryoprotectors: oligosaccharides, including the most frequently used, sucrose and trehalose [39], and high molecular weight polymer compounds, such as ficoll, albumin, and polyvinylpyrrolidone [40] (Table 1). Due to their high molecular weight, exocellular cryoprotectors cannot penetrate cell membranes, unlike DMSO, glycerol, and the other low molecular weight cryoprotectors. Since exocellular cryoprotectors are located outside the cell, they have several advantages over the endocellular alternatives. Their impact on cells is gentler due to their lower toxicity, and they can be easily removed after thawing, during the washing process. Exocellular cryoprotectors are often used in combination with endocellular cryoprotectors. Such combination of cryoprotectors allows for increasing the survival rate of biological objects, compensating for the disadvantages of one category with the advantages of the other [38,41].

### 2.2. Dimethyl Sulfoxide (DMSO)

Despite the fact that there exists quite a large number of substances with cryoprotective characteristics, medicine and laboratory practices typically operate with merely a dozen such compounds. The most frequently used cryopreservation solutions are DMSO-containing freezing media. DMSO became globally accepted in the 1960s. It was allowed for use in clinical medicine, and since that moment DMSO has become the preferred cryoprotector in many countries [42]. It should be noted that in some countries, for example, Russia, DMSO remains the sole officially approved cryoprotector for cell-based products for clinical use. DMSO has good penetration capacity in relation to the living cells of animals and humans. It is also important that relatively low concentrations of this cryoprotector—ranging from 5 to 15%—are sufficient to fully protect cells from damage during freezing [36,43]. This aspect enables minimization of the cytotoxic effect of such low molecular weight cryoprotectors.

For the cryopreservation of stem cells, DMSO solution at a finite concentration of 10% is most often used [44,45,46,47,48]. The remaining 90% is accounted for by various other components and combinations thereof. For example, Solodeev, I. et al. [44] used a cryopreservation medium containing 90% fetal bovine serum and 10% DMSO. Shaik, S. et al. [45] used a cryoprotective medium containing 10% DMSO in a phosphate buffer solution for the cryopreservation of stem cells taken from healthy donor lipoaspirate. As in the previous study, Bárcia, R.N. et al. [48] used 10% DMSO and 90% phosphate buffer for the cryopreservation of umbilical cord MSCs (UC-MSCs). Chabot, D. et al. [47] used a solution containing 10% DMSO, 0.9% dextran, and 5% HSA (human serum albumin) to freeze UC-MSCs. The above-mentioned authors confirmed that the thawed cells fissioned well in culture and were able to generate colony-forming units and to express surface markers. Their phenotypes were similar to non-frozen cultures [44], and cryostorage had no significant impact on the potential for adipogenic and osteogenic cell differentiation [45]. Fresh MSCs, which were not subjected to cryopreservation, and cryopreserved cells showed comparable viability, functionality, and integrity [47]. Cells retained their immunomodulating ability and angiogenic potential after cryopreservation [48]. The authors suggested that in the case of administering cryopreserved cells and fresh cells in similar doses, both types would have similar clinical efficacy [44,48].

The above-mentioned literature sources indicate that the properties of MSCs after freezing with DMSO did not change significantly and remained equal to their non-frozen counterparts. However, there are other studies demonstrating that the thawed cells are inferior in quality to cells not subjected to cryopreservation. For instance, Francois, M. et al. [49] specified that freezing with DMSO caused changes in the phenotype and proliferation of MSCs. Early passages of human brain MSCs were frozen in α-MEM, 30% fetal bovine serum, and 5% dimethyl sulfoxide. Cell viability analysis showed that the percentage of living cells in fresh cultured MSCs was 92% and 91%. Whereas the percentage of living cells in cryopreserved cells decreased to 61% and 44%. The authors demonstrated that freshly thawed MSCs activated heat shock proteins and were unable to suppress T-cell proliferation in vitro due to disruption of indoleamine 2,3-dioxygenase (IDO) induction in response to interferon (IFN)-γ. Such observations provide some insight into the reasons for the mismatch between the results of the preclinical and clinical efficacy of MSCs in the treatment of immune diseases. The immunosuppressive activity, sensitivity to IFN-γ, and IDO induction were completely restored within 24 h of acclimatization after thawing of the MSC culture [49].

Similar results were obtained by Moll, G. et al. [50] with MSCs isolated from bone marrow aspirates of healthy donors. In order to compare fresh and frozen MSCs, the cells were divided into two equal fractions. One fraction was kept at +4 °C until infusion, the other was frozen with a 10% DMSO solution. Immediately before the experiment, cryopreserved MSCs were thawed and washed twice with the phosphate buffer solution. The authors showed that the cryopreserved MSCs had a lower therapeutic activity as compared to the fresh cells due to an increased manifestation of proapoptotic properties and impaired immunosuppressive activity. Patients, who received fresh cells at an early passage, demonstrated a response rate of 100%, which was twice that registered in the group of patients who received cells immediately after thawing. The authors suggested that further use of freshly thawed cells requires a phase of recovery or acclimatization of the MSCs after thawing [50].

Based on these and other similar studies [41,51,52], one can conclude that the decrease in cell quality after cryopreservation can be reversible and, as a rule, the quality is restored during the subsequent cultivation. The reversible adverse effect of cryopreservation is most likely related to the cells’ responses to heat shock stress during thawing. Temporary repression of non-vital expressed genes during their stress response allows cells to prioritize survival over the restoration of their functional properties [49]. These results indicate that therapeutic MSCs must be restored in culture before clinical use.

### 2.3. Reduction of the Cytotoxic Effect of Cryoprotective Media Containing DMSO

To avoid the negative impact of DMSO on the functional activity of stem cells, various combinations of non-cytotoxic biocompatible substances are used together with DMSO. For instance, Wang, J. et al. [53] studied the impact of the cryoprotective protein AavLEA1 on MSC survival using 0%, 2%, 5%, and 10% dimethyl sulfoxide solution. The authors found that the survival of MSCs amended with the AavLEA1 protein was significantly higher than that of cells cryopreserved with a low concentration of DMSO solution, and the rate of apoptosis and necrosis decreased, respectively [53].

Pollock, K. et al. [54] used media consisting of 60% plasmaline, 20%–25% HSA, and 20% DMSO (the finite concentration of DMSO was 10% on a volume basis) as cryoprotective agents. The total number of MSCs, their viability, apoptosis, and aging were assessed during 6 passages in ex vivo culture. The cells were frozen in a freezer at a controlled rate (1 °C/min). After completion of the freezing protocol, the samples were stored in liquid nitrogen for 30–45 days and then water bath thawed at 37 °C. Some of the cells were immediately analyzed for viability and aging, whereas the remaining cells were plated and analyzed after 48 h. The viability of the samples before freezing and after thawing did not differ significantly. The trends in the samples after thawing were consistent with those in the populations before freezing (expression of beta-galactosidase as an indicator of aging, percentage of apoptosis) [54].

**Figure 3:**
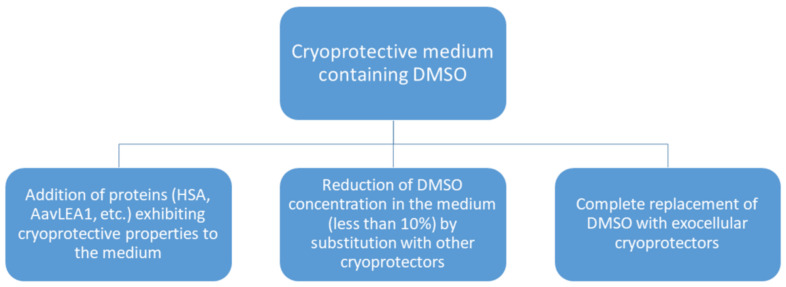
Ways to reduce the cytotoxicity of cryoprotective media containing DMSO.

An alternative approach to increase the cryopreservation efficacy is to find ways to reduce the DMSO concentration in order to lower its cytotoxic effect (Figure 3). For example, Svalgaard, J.D. et al. [55] used pentoisomaltose, a synthetic carbohydrate, originally created as an alternative to DMSO, for these purposes. The authors compared pentoisomaltose-based freezing media containing 1% or 2% DMSO with commercial CryoStor media (Biolife Solutions, Bothell, WA, USA) based on dextran-40 with 2% and 10% DMSO concentrations. An internal standard freezing medium with 10% DMSO was used as a control. The results showed that adding pentoisomaltose to the freezing medium ensures a better cell-based product than using a medium based on dextran-40. Here, the DMSO concentration could be reduced to 1% without loss of the cryoprotective properties [55].

Another way to improve the efficacy of cryofreezing is the total replacement of DMSO with exocellular cryoprotectors, for example, with oligosaccharides [39]. Trehalose, a non-reducing disaccharide known for its unique physicochemical properties, can be used as a cryoprotective agent and as an alternative to DMSO. Due to its interaction with plasma membrane phospholipids, trehalose contributes to the stabilization of cryopreserved cells by reducing osmotic stress [56]. For instance, Roato, I. et al. [10] compared freezing media containing 10% DMSO and 0.35 M trehalose, respectively. However, the results showed that dimethyl sulfoxide outmatched trehalose as a cryoprotector: cultures of fatty MSCs cryopreserved in DMSO demonstrated faster growth and better morphology than cultures cryopreserved with trehalose. It should be noted that exocellular cryoprotectors are often used as additional components in solutions of endocellular cryoprotectors, as the use of exocellular cryoprotectors alone may not be sufficient, as was proved by Roato, I. et al. [10].

Freimark, D. et al. [57] investigated various combinations of ectoine and proline as potential cryoprotectors. The freezing medium in the experimental group contained basal medium (PBS with Ca^2+^, Mg^2+^, and methylcellulose) with various concentrations of ectoine/proline. The control groups used basal freezing medium supplemented with 10% DMSO (positive control) or pure basal medium (negative control). The use of the freezing medium with lower proline (1%) and higher ectoine (10%) showed promising results, although the highest survival rate was achieved with the commercial Biofreeze medium [57].

### 2.4. Commercially Available Cryoprotective Media

In addition to in-house freezing media formulations available in virtually every laboratory, there are commercially available ready-to-use cryoprotective media. The Biofreeze commercial medium (Biochrom, Berlin, Germany) has already been mentioned above. Another example of a commercial cell freezing medium is Bambanker (Lymphotec, Tokyo, Japan), offered by a Japanese company and originally developed for the company’s own projects. For example, Huang, Y.H. et al. successfully used Bambanker for the cryopreservation of stem cells isolated from adult dental pulp [58]. Hoang, V.T. et al. [59] used CryoStor CS10 commercial serum-free, xenogenic-free medium (STEMCELL Technologies, Vancouver, BC, Canada) for the long-term storage of MSCs isolated from umbilical cord, bone marrow, and adipose tissues. Cryostorage was arranged in an automated Brooks system (Brooks Life Sciences, Chelmsford, MA, USA) in liquid nitrogen. The authors demonstrated that the quality of the MSCs remained unchanged after cryopreservation and in vitro cultivation. The cells retained the expression of biomarkers and a normal karyotype, were capable of colony formation, and successfully differentiated into osteogenic, adipogenic, and chondrogenic lines [59].

The composition of commercial media, in general, is not disclosed by manufacturers; however, those media the composition of which has been partially disclosed, often contain DMSO, for example, CryoStor^®^ CS10 contains 10% DMSO [60].

The advantages of ready-made commercial cryoprotectors are undeniable, they do not require dilution or the addition of ad-hoc components, which simplifies working with them to a major extent. They are standard-based, and their composition is constant regardless of the batch. Many commercial environments do not require staged freezing, so this saves a lot of time.

Despite the existing variety of cryoprotectors and cryoprotective media, the development of new and more effective cryoprotectors is ongoing. One of the modern directions in improvement of cryopreservation techniques includes studies aimed at creating multicomponent media, the formulations of which, along with the cryoprotectors, include “restoring” additives (carbohydrates, plasma proteins, biologically active compounds, salts, etc.) in order to maintain energy metabolism in the thawed cells, to reduce toxicity and to eliminate other side effects. At present, innovations in synthetic polymer chemistry facilitate the design of the molecular architectures of complex cryoprotectors, allowing for the development of synthetic substances (e.g., zwitterionic polymers such as 2-methacryloyloxyethylphosphorylcholine (MPC)) with functions similar to antifreeze proteins [56].

## 3. Cryopreservation of MSC Suspensions

**Figure 4:**
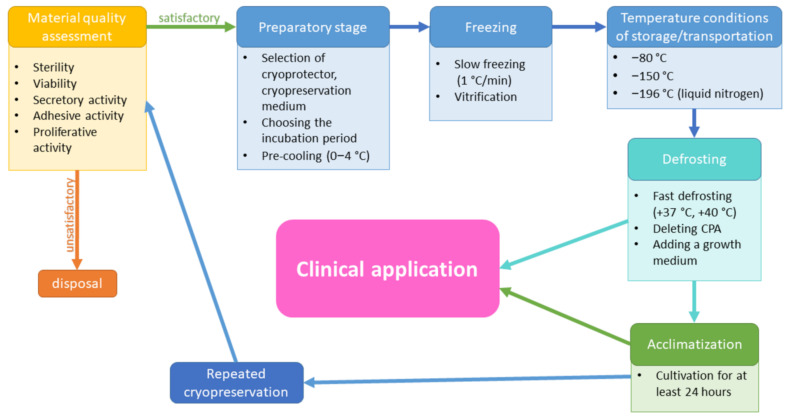
The main stages of a cryopreservation protocol.

Cryopreservation can be considered successful only if the cell culture can be restored to physiological functioning with an insignificant loss of cell viability and functional activity [61]. At present, there are many MSC cryopreservation protocols that have been developed for the majority of areas of research and medicine [62]. However, differences at the level of protocols lead to different efficacies of cryopreservation, which cause variability in the quality of the cell-based products after cryopreservation. Cryopreservation protocols are usually multi-phase and include a number of successive steps: preliminary cell preparation for cryopreservation, freezing of the cells, the control of cryopreservation and potential transportation of the frozen cell-based products, with regard to the compliance with a certain temperature regime, thawing, and acclimatization of the cells (Figure 4). Each phase allows many modifications. This makes standardization impossible and, thus, complicates the clinical use of cell-based products.

### 3.1. Preparatory Phase

As temperature decrease provokes cells stress reactions, a mandatory step in cryopreservation includes preliminary preparation of the cells before freezing, aimed at minimizing damage and preventing their death. The success of this procedure, as well as of cryopreservation in general, will largely depend on the quality of the material to be frozen: cells prepared for cryopreservation must be in their optimal condition, with high viability, to ensure maximum survival rates during freezing and after thawing [54,63]. Therefore, culture quality assessment is an additional step that should precede the cryopreservation preparatory phase. In general, assessment of the quality of cell-based material is limited to assessment of cell viability; however, even viable cells may show decreased secretory, adhesion, or proliferative activity. Thus, to obtain the highest quality product after cryopreservation, one should conduct a comprehensive preliminary assessment of the culture, including not only viability, but also functional activity [64].

It should also be noted that the criteria for assessment of the culture quality before cryopreservation often do not coincide with the criteria applied after thawing. As a result, it is not always possible to assess the reasons for any loss of culture quality. On the one hand, cryopreservation could cause deterioration in the quality of the cell material, but on the other hand, the culture quality could have been low initially, where an insufficient preliminary quality assessment prevented this from being detected. Consequently, any culture quality assessment before and after cryopreservation should be conducted in line with the same parameters, thus ensuring a correct assessment of the impact of cryopreservation on the culture [65].

The preparatory phase includes replacement of the growth medium with the cryopreservation medium containing the cryoprotector and subsequent incubation at positive temperatures. Thus, the cryoprotector can replace the liquid before freezing. Differences of this phase depend on the type of cryoprotector used and may vary individually, based on the protocol.

For instance, after adding the penetrating CPA, incubation at physiological temperatures is recommended [37]. This is due to the fact that the rate of cryoprotector penetration through the cell membrane is decreased at reduced temperatures, which means that the amount of CPA entering the cells depends on adhering to the correct incubation conditions. However, cooling not only reduces the penetrating ability of the cryoprotector, but also lowers its cytotoxicity; therefore, in order to avoid CPA-induced toxic damage to cells, one can consider lowering the incubation temperature if its duration is increased [66]. By contrast, exocellular cryoprotectors do not require time to pass through the cell membrane as their action is based on hindering the growth of crystals around the cells, and if these are used, the phase of pre-incubation before freezing can be avoided [67].

It is also should be noted that, regardless of the type of cryoprotector used, most protocols include a phase of sample cooling at low positive temperatures (0–4 °C) immediately before freezing. This is to overcome the extra stress that an abrupt decrease in temperature can add to the sample, which could subsequently affect the recovery of the cells after thawing [66,67].

### 3.2. Freezing Rate

One of the major aspects influencing cryopreservation success is the freezing rate. During cooling, the balance of osmotic pressure in the cells is upset due to dehydration. Additionally, there is always a risk of intracellular and extracellular ice buildup, which can cause mechanical damage to the cells [62]. Therefore, the freezing rate must be fast enough to avoid the imbalance of dissolved substances and electrolytes, but slow enough to prevent ice crystal buildup [68]. It should be noted that the choice of cooling rate during freezing is also influenced by biophysical parameters specific for each cell type, as well as the type and concentration of the cryoprotector [28,69].

The most commonly used technique is programmed cell freezing, which includes a controlled cooling rate of about 1 °C/min and the availability of less than 1.0 M CPA in the freezing medium. The advantages of this protocol include low cytotoxicity and insignificant osmotic effects due to the low concentration of the cryoprotector, as well as the relative simplicity of the protocol. However, slow cooling increases the risk of extracellular ice buildup and, thus, of mechanical damage to the cells. At the same time, additional equipment is required to implement this technique. To do so, program freezers with an adjustable cooling rate are used. However, they are quite expensive and require tailored maintenance as liquid nitrogen serves as the cooling agent [28].

An alternative to slow cooling is vitrification, being the process of converting liquid into a vitreous structure when exposed to extremely low cryogenic temperatures (liquid nitrogen temperature is −196 °C). As cell freezing occurs quickly in this case, ice crystals do not have time to build up, thus the risk of mechanical damage to the cells is minimized. However, this method requires adding CPA at high concentrations (up to 8 M), which then provokes osmotic imbalance at thawing; the protocol technique also requires good manipulation skills [70].

### 3.3. Temperature Conditions

Along with the problem of choosing an optimal freezing rate, the issue of the cryopreservation temperature regime, which is directly related to the technical equipment available in medical institutions, is of equal importance. The storage of cell-based products in liquid nitrogen at a temperature of −196 °C is considered optimal, since the cells are in metabolic stasis at this temperature [29]. However, the cost and complexity of liquid nitrogen supplies on demand, as well as the need for separate specially equipped premises for such a cryobank, force many organizations to look for alternatives [69].

Storage of samples at a temperature of approximately −80 °C is more accessible and most often applied in clinical practice. For instance, it has been confirmed that blood samples can be successfully stored at this temperature for up to 180 days [71,72]. However, in the case of MSCs and tissue samples, the duration of cryopreservation can be up to 10 years, but this temperature regime does not allow for such a long period of sample preservation [10,73]. A number of studies have demonstrated that with a short storage period (e.g., several months) at −80 °C or at −196 °C, the viability of the thawed MSCs is comparable [10,74,75]. In the case of longer cryopreservation (e.g., more than 1 year), it is possible to use freezers that can maintain a stable temperature of about −150 °C, which is an acceptable alternative to liquid nitrogen [69].

It should be noted that even for a short cell storage period, the use of freezers with a temperature of less than −80 °C is rare. In some cases, successive freezing of the MSC suspension at −20 °C is applied, and then there is a transfer to storage at −80 °C [76,77].

It must be highlighted that any given temperature regime must be maintained throughout the storage period, including periods when the sample container is removed from the freezer for audit or transportation. Thermal transition processes when samples are brought into or removed from a freezer can significantly affect the cells’ condition after thawing. These can be minimized by separating the main biobank stocks from areas with frequent activity, as well as by transporting samples in tightly closed containers that maintain the required temperature [69,78,79].

### 3.4. Thawing

The thawing process is another critical step that determines the success of cryopreservation. The most frequently used protocol includes water bath thawing at +37 °C until the ice crystals completely disappear [80,81,82]. Due to rapid thawing (at approximately 100 °C/min), there is no liquid recrystallization, thereby avoiding cell damage at this stage and during removal from cryostorage, minimizing the risk of cell death [78]. Variations of this phase of the thawing protocol are practically non-existent, only being associated with the equipment used (water bath or thermostat) [83]. It should be noted that, previously, thawing in a freezer at a controlled rate and temperature change of 10 °C/min was used, but this protocol was effective only for MSC cryopreservation from early passages [84].

After thawing, it is necessary to remove the cryoprotector from the cell suspension by diluting it with the prepared growth medium followed by centrifugation (several repeated cycles are allowed). In the case of vitrification, when high cryoprotector concentrations have been used, the nutrient medium can be amended with sucrose, which reduces the osmotic pressure differences [70]. After supernatant removal, the cells are resuspended in the fresh growth medium and cultured, or the condition of the cells is assessed immediately, subject to their subsequent use [67,81,85].

### 3.5. Acclimatization

Several studies have demonstrated that, after thawing, MSCs must be acclimatized, meaning that they cannot be used immediately after thawing, but should be subjected to a certain period of cultivation. This promotes better culture recovery and also increases the amount of cell material [86]. The importance of acclimatization was clearly shown by Bahsoun, S. et al. [87]. The authors used a protocol that included water bath thawing at a temperature of +40 °C until the ice crystals completely disappeared, after which the post-cryoconservation state of marrowy MSCs was assessed 0, 2, 4, and 24 h after thawing. The results showed a decrease in viability, metabolic activity, and adhesion potential of the MSCs in the first 4 h after thawing. After 24 h, the apoptosis level decreased, and viability was restored; however, MSC activity remained lower than for that of fresh cells. Thus, even a 24-h expansion may not be enough to fully restore the MSCs’ therapeutic potential [87].

### 3.6. Repeated Cryopreservation

The issue of repeated cryopreservation, as well as the effects of multiple freeze–thaw cycles is of equal importance. Recent research shows that early passage cultures can be frozen and thawed up to two times without any loss of activity or functionality [88]. However, four or more freeze–thaw cycles cause both early aging and a decrease in the MSCs’ proliferative activity, which may be related to accumulated stress impact from the multiple cryopreservations. Studies show that already during the third or fourth cycle, cell doubling time significantly increases, morphology starts to change, and immunomodulatory efficacy in vitro decreases [46,80].

It should be recognized that the development of an optimal, standard cryopreservation protocol seems to be almost impossible as all laboratories have different research objectives, materials, equipment, and reagents, and therefore use the protocols that are most applicable for them. However, standardized quality control may be the key to optimizing biobanking and correctly comparing cell-based products [89].

Moreover, it should also be taken into account that various cell types can differ in their membrane permeability and surface-to-volume ratios, which can lead to differences in their response to freezing [69]. For example, differences in response to cryopreservation were noted even in different types of leukocytes [90]. Thus, for each type of MSC, its own optimal protocol should be developed.

## 4. Features of Cryopreservation of Pluripotent Embryonic Stem Cells and Induced Pluripotent Stem Cells

A special position among stem cells is held by pluripotent cells—pluripotent embryonic cells (ESs) and induced pluripotent stem cells (iPSCs). They are closely related to MSCs. At the same time, compared to other types of stem cell, they have a number of distinctive features invariably attracting researchers’ interest. Thus, human pluripotent embryonic stem cells, unlike MSCs, have an unlimited capacity for self-renewal and, in culture, retain their pluripotent ability to differentiate into cells of all three germ layers. It is noteworthy that human pluripotent embryonic stem cells (hESs) differ from MSCs phenotypically. The set of markers characteristic of hESs include the stage-specific embryonic antigens SSEA-3 and SSEA-4, TRA-1-60, TRA-1-81, CD9 and CD133, Thy-1 (CD90), MHC class 1 and intracellular transcription factor Oct 3/4 [91]. hESs also show high levels of alkaline phosphatase and telomerase activity [69]. In 2006, Takahashi, K. and Yamanaka, S. [92] successfully reprogrammed differentiated adult stem cells and T-lymphocytes into a pluripotent state by fusing them with human embryonic stem cells [93]. These cells were called “induced pluripotent stem cells”. iPSCs exhibit gene expressions, epigenetic profiles, and differentiation potentials similar to hESs [94]. The generation of such PSCs spurred the discovery of the true potential of hESs greatly enabling the expansion of research while eliminating the ethical problems associated with obtaining new embryonic cells [95].

One of the problems when working with ESs and iPSCs is how the behavior of such cells in culture impacts the possibilities of their effective cryopreservation. Unlike adult stem cells, which grow in culture as attached monolayers, ESs and iPSCs usually grow in an undifferentiated state, as colonies (aggregates). These epiblast-type colonies are characteristic not only for human cells, but also for cells from other highly evolved animals like monkeys and pigs [96]. The cells in these aggregates are closely related and there are typically 3 × 10^4^ to 5 × 10^4^ cells per colony [69]. One of the key features of cells in colonies is their tendency to lose viability when attempts are made to separate the cells from each other. As a consequence, epiblast stem cells have, historically, been passaged and cryopreserved as aggregates.

The following advantages of aggregates are reported in the literature, but they can also be considered as disadvantages at the same time: (1) the intercellular contacts with neighboring cells promote cell survival, (2) the freezing/thawing of aggregates usually leads to a faster recovery compared to that for single cells, because the single cells need more time to form aggregates again. However, the heterogeneity of aggregate sizes leads to different cryoprotectant penetration into the aggregate nucleus during freezing, which in turn affect cell viability after thawing [97]. Data have been reported regarding the optimal cell cluster size for cryopreservation being around 100–500 cells [98]. If the aggregate size exceeds these values, only a small fraction of the cells survive cryopreservation [96].

Traditional methods of cell cryopreservation with slow freezing procedures [96,99] and vitrification [100,101,102] have been widely used for the storage and transport of ESs and iPSCs. However, these two methods have disadvantages related to the low percentage of viable cells preserved, low recovery rates, and high levels of spontaneous differentiation after thawing [103,104,105,106]. Closed cell cryopreservation systems like the CryoLogic Vitrification Method (CVM) have therefore been developed recently to store and transport embryonic cells. They allow the maintenance of an optimal recovery rate and preserve a high stemness level and the differentiation potential of hiPSCs [102].

Thus, cryopreservation, a process largely developed for MSCs, has only recently begun to be studied for ES and iPS cells. Improvements of traditional slow-cooling/vitrification protocols and the new container systems being developed are likely to produce systems compatible with the requirements of GLP and GMP, control, automation and scalability. Although the field of ES/iPSC cryopreservation has advanced considerably, it still faces many challenges. Solving them will broaden the prospects for the scientific and clinical applications of ESs and iPSCs in regenerative medicine [94].

## 5. Cryopreservation of Tissue Specimen of MSC Sources

**Figure 5:**
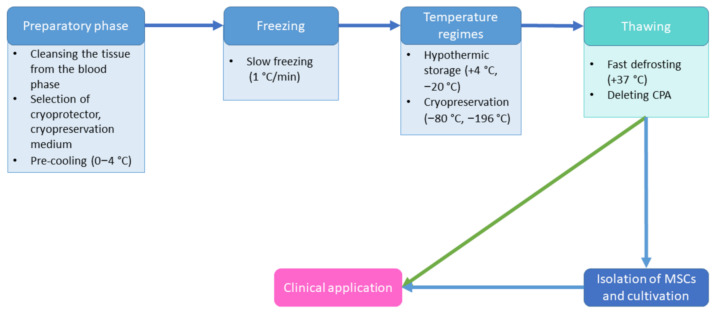
Generalized scheme of the protocol for cryopreservation of adipose tissue.

Quite often, the object of biobanking is not the MSC suspension but the tissue, for example, adipose tissue in the form of lipoaspirate. Several studies have demonstrated the efficacy of cryopreserved adipose tissue as a source of stem cells ready for transplantation [107,108,109]. For instance, it has been confirmed that MSCs isolated from cryopreserved adipose tissue are viable, and their functional activity is almost comparable to freshly isolated MSCs [107]. Moreover, in cells isolated after tissue cryopreservation, an increase in the expression of stromal and adipogenic markers is seen against a decrease in the expression of hematopoietic markers, while the MSCs’ morphology is similar to that of freshly isolated cells [108]. It should be noted that the cell isolation step can be skipped, and then the adipose tissue, itself, will be used as a graft. However, after thawing and regardless of the cryopreservation duration, only about 67% of the initial tissue volume on average is restored, so one should always freeze a larger tissue volume than is required for use [109]. The cryopreservation protocols for adipose tissue include the same steps as those for cell suspensions, but, despite multiple similarities, they do have their own specifics (Figure 5).

### 5.1. Preparatory Phase

The preparation of adipose tissue for cryopreservation includes cleaning the blood from the tissue by sedimentation and/or washing with a buffer solution, cryoprotector addition, and pre-cooling. The last of these is of special importance as the tissue has a larger volume compared to the cell suspension, which can result in a lack of uniform cooling. Abrupt cooling of such samples to ultra-low temperatures can lead to cell death and, as a result, to grafts having low viability after thawing [83]. Therefore, prior to freezing, the tissue is usually first cooled at low positive temperatures (4 °C) and only subsequently at negative temperatures (−20 °C) [110,111].

As with cell suspensions, the most commonly used cryoprotector is DMSO in combination with fetal bovine serum (FBS) or calf serum (CS). Frequently, in order to avoid zoonotic infection during adipose tissue transplantation, FBS and CS are replaced with donor or patient plasma components, such as platelet-poor plasma or serum albumin [112,113].

The concentration of cryoprotectors may vary in different protocols. For instance, during short-term storage (up to 3 months) Massiah, G. et al. [112] showed the same efficacy for DMSO concentrations of 5% and 10%, and for freezing in a medium without a cryoprotector. However, the viability of the thawed samples was only tested ex vivo. It should be taken into account that their deferred studies in vivo may show completely different results [112]. Gu L. et al. [110] used various concentrations of fetal calf serum (15% and 30%) in combination with 7.5% DMSO for adipose tissue cryopreservation. The beneficial effect on the viability of the thawed tissue of a large amount of serum (30%) in the cryoprotective mixture was demonstrated [110].

As in the case of the cryopreservation of MSC suspensions, a combination of endo- and exocellular cryoprotectors is used for tissue freezing. For example, in a systematic review of the literature, Crowley, C.A. et al. [114] confirmed high efficacy of the combined use of DMSO and trehalose. It was also noted that trehalose can be used as an independent cryoprotector, the efficacy of which can be increased by delivering the trehalose into the cells.

At present, increasing attention is being given to the development of non-toxic cryoprotectors, in particular, for the biobanking of adipose tissue. Such cryoprotectors include glycerol, which demonstrates high efficacy (at an optimal concentration of 70%) in comparison with DMSO and trehalose. It should be noted that adipose tissue cryopreservation with the use of glycerol as a cryoprotector was tested only for short periods (up to 1 month). Its efficacy over longer periods of tissue cryopreservation has not yet been confirmed [115].

The freezing of adipose tissue without a cryoprotective agent has been reported in some studies [116,117]. It was noted that this cryopreservation option is not significantly inferior in efficacy to freezing with a cryoprotective medium and may be better in terms of the time spent on the procedure. It is assumed that the reason for the survival of adipose tissue cells after cryopreservation without a cryoprotector, apart from the absence of any stress impact on the cells due to the cryoprotective agent itself, may be the cells’ availability in the structure of the natural matrix—adipose tissue. Nevertheless, one can still get a larger number of viable MSCs when using a cryoprotector [118,119].

Thus, the selection of an optimal composition of the cryoprotective mixture for adipose tissue cryopreservation still remains a topical issue and provides many opportunities for research.

### 5.2. Freezing Rate

As with the cryopreservation of cell suspensions, the adipose tissue freezing rate can have a significant impact on the efficacy of the cryopreservation process in general. As mentioned above, compared to cell suspensions, a tissue sample has a larger volume and higher density, and thus its cooling is non-uniform. Due to such non-uniform sample cooling, cryopreservation of adipose tissue is conducted mainly by means of slow freezing (about 1 °C/min) [10,73,118,119]. The use of vitrification for the cryopreservation of adipose tissue is almost absent from the literature.

### 5.3. Temperature Regimes

Choosing an optimal temperature regime for adipose tissue cryopreservation is as important as for cell suspension cryopreservation, and is directly related to the cryopreservation duration. For instance, Kim D.Y. et al. [120] obtained the largest number of viable cells from tissue samples stored at a temperature of 4 °C; however, their functional activity was rather low, and the overall storage period of the samples was 1 week at a maximum. MSCs isolated from tissues stored at −80°C were viable and functional. However, it turned out to be impossible to isolate viable cells from samples stored at −20 °C and −196 °C [120]. It should be noted that the optimal time for the hypothermic storage of adipose tissue (4 °C) is up to 24 h [118].

In another study, the authors demonstrated that in the case of longer cryopreservation periods (up to 1 month), storage of adipose tissue samples at −80 °C and at −196 °C showed the same efficacy, and that the MSCs isolated after thawing of these samples had efficacies comparable to freshly isolated cells [10]. As in the previous study, the authors did not manage to isolate viable cells from adipose tissue stored at −20 °C [73].

It should be noted that cryopreservation at −80 °C allows the preservation of adipose tissue samples for up to 3 months, even without the use of a cryoprotector [118]. However, it may be necessary to store adipose tissue for longer periods of time, such as a year or several years. Unfortunately, there are presently very few studies on such long-term tissue cryopreservation.

### 5.4. Thawing

As with the cryopreservation of cell suspensions, adipose tissue cryopreservation is most frequently conducted in line with the principle of slow freezing and fast thawing. The thawing protocol usually involves the water bath thawing of samples at 37 °C and removal of the cryoprotector by washing with a buffer solution, followed by stem cell isolation [10,73,107,110,111,118,119].

In general, tissue cryopreservation is characterized by simpler sample preparation, as it does not require time for cell cultivation and preliminary assessment of the culture quality. Cells isolated after cryopreservation are comparable in properties with cells freshly isolated from native tissue, although they are fewer in number. Moreover, it is possible not to have to isolate stem cells from the adipose tissue, but to use it as a single graft. In this case, it has been observed that the adipose tissue can act as a matrix for new cells recruited from the recipient’s tissues, and that this can increase the efficacy of therapy [116,118]. It should be noted that many aspects of adipose tissue cryopreservation have not yet been studied sufficiently and require additional detailed research. There are very few studies on the cryopreservation of bone marrow tissue, as the following are most frequently used for cryopreservation: bone marrow MSC suspensions [98], the mononuclear cell fraction [120], or concentrates of bone marrow aspirate [121]. Despite the fact that a study in mice showed a high efficacy for bone marrow cryopreservation in the form of a tissue flap [122], it is very difficult to apply this approach to patients, as the material for cryopreservation can only be obtained during total orthopedic surgical procedures [123].

A number of studies have shown successful results for the cryopreservation of human umbilical cord tissue, one of the fairly accessible sources of MSCs. According to the data presented by Harutyunyan, I. et al. [124], at least ten cryobanks around the world, including in the US, UK, Australia, and South Africa, offer human umbilical cord tissue preservation services. Cryopreservation protocols are based on slow freezing (1–2 °C/min) followed by storage of the tissue samples at −80 °C or in liquid nitrogen vapor. To defrost the samples, thawing at +37 °C is most often used, then the cryoprotectant is removed by washing with buffer solution, and the stem cells are extracted immediately. However, despite such international experience of the successful preservation of human umbilical cord tissue, common problems characteristic of the cryopreservation protocols of MSCs and MSC-rich tissues are also inherent in the protocols used for umbilical cord blood tissue. For example, most protocols use DMSO as the cryoprotectant, but the cytotoxic effect of this reduces the viability of the umbilical cord tissue cells. Therefore, many of the cryopreservation studies of human umbilical cord tissue are aimed at searching for an alternative cryoprotectant. Thus, ethylene glycol, glycerol, 1,2-propanediol, and commercial ready-to-use cryoprotective media have been considered, as these show better results compared to DMSO in terms of the preservation of living cells in the frozen tissue, the early start of migration of these cells from thawed explants, and the overall efficiency of such multipotent stromal cells [125,126,127,128].

## 6. Cryopreservation of Three-Dimensional Biomedical Cell-Based Products

Nowadays, the successful development of regenerative medicine is closely linked to the use of complex biomedical cell-based products with three-dimensional structures instead of individual cells (such as the transplants and constructs). From a clinical perspective, it is very important to learn how to cryofreeze multicomponent cell-based products rather than individual cells. For instance, during the development of artificial organs, a promising approach in manufacturing potential replacements for artificial organs is the cryopreservation of the whole structure and not just its separate parts.

One of the first steps in the transition from the cryopreservation of simple cell suspensions to the cryopreservation of three-dimensional objects was the freezing of cells encaged in polymer capsules. For instance, to overcome the difficulties of freezing adhesive monolayers and to simulate a three-dimensional structure, MSCs were cryopreserved in an encapsulated form in alginate microspheres (AMSs). AMSs are permeable to oxygen, nutrients, and signaling molecules, thus enhancing the viability and functioning of the encapsulated cells. AMSs can act as a barrier to immune responses in transplantation while supporting both cell proliferation and differentiation. Moreover, alginate is hygroscopic and, by absorbing water, it can prevent the buildup of large ice crystals during freezing; that is, the alginate hydrogel, itself, may have cryoprotective properties. In his study, Pravdyuk, A.I. [129] cryopreserved bone marrow MSCs encapsulated in alginate in 5% or 10% DMSO using three different cryopreservation protocols. After cryopreservation with three-step slow cooling with controlled ice nucleation, the MSCs encapsulated in alginate microspheres could reach multilinear differentiation directed towards osteogenic, adipogenic, and chondrogenic lines. The author concluded that cryobanking of microsphere-encapsulated MSCs is quite manageable and can be used for future projects in regenerative medicine.

The next step in the development of cryopreservation of three-dimensional structures is research on the preservation of scaffolds with encapsulated cells. Katsen-Globa, A. et al. [130] investigated the attachment and distribution of MSCs inside an alginate–gelatin scaffold before cryopreservation. MSCs obtained by UC-MSC isolation were plated into a scaffold, and the complex (cells and scaffold) was kept in 10% DMSO for a period of 5 min at 4 °C, followed by cooling to −80 °C; here it was kept overnight before immersion in liquid nitrogen. The authors demonstrated that cell viability, cell contacts, membrane integrity, and MSC mobility were comparable with the control unfrozen MSCs [130].

Diaz-Dussan, D. et al. [56] assessed the cryoprotective efficacy of trehalose-based hydrogels using mammalian cells: cervical cancer cells (HeLa), prostate cancer cells (PC3), and skin fibroblasts (normal cell line). The authors reported on the synthesis of the hydrogel based on trehalose and its use as a both a cryoprotector and a three-dimensional cell scaffold. Trehalose was shown to be an active protein stabilizer, minimizing protein aggregation and increasing activity when exposed to higher temperature, pH changes, agitation, and drying. The studies demonstrated that trehalose-based scaffolds act as cryoprotective agents that can significantly influence the buildup and growth of ice crystals, mitigating physical damage to the cells during freezing and thawing, thus improving the cryopreservation results. The MSCs’ viability, proliferation, and differentiation, as well as the mechanical integrity of the scaffold, were similar to those of their counterparts which had not been subjected to cryopreservation [56].

Similar results confirming that scaffolds can exhibit cryoprotective properties and thus allow preservation of the cell-based component were obtained by Nagao, M. et al. [131]. The authors investigated the efficacy of biocompatible triblock copolymers both as gelling agents and as cryoprotectors. A temperature sensitive and zwitterionic triblock copolymer, PDEGMA113-b-PMPC243-b-PDEGMA113, was synthesized by atom transfer radical polymerization. Both 2D and 3D culture studies confirmed that this hydrogel could be used as a matrix/scaffold for a cell culture in vitro. The authors confirmed the good biocompatibility and low cytotoxicity of the hydrogel. It was shown that the cells retained high viability after freezing with 3% and 15% polymer solution, which was similar to the results of the control, i.e., cells frozen with 10% DMSO solution. It should be noted that the integrity of the cell membrane after thawing was over 95%, which indicates a great potential for using this triblock copolymer as a cryoprotector [131].

Mutsenko, V. et al. [132] developed a combined protocol for the cryopreservation of tissue-engineered constructs. Porous 3D scaffolds based on mineralized collagen suspension plated with amniotic MSCs of *Callithrix jacchus* were subjected to 24-h pre-freezing with 100 mM sucrose in the culture medium, after which a cryoprotector was added dropwise to the samples to obtain a finite concentration of 10% DMSO and 20% FBS. Some samples were amended with 300 mM sucrose. Then, the samples were left on ice for 15 min, after which excess cryoprotective mixture was taken out. After that, the scaffolds were frozen to −152 °C and stored for 5 days at this temperature, and then thawed. Assessment of the cell viability in the scaffolds showed that the sucrose pre-treatment had reduced the development of osmotic stress during freezing. Moreover, cryopreservation according to the protocol, including the addition of sucrose into the cryomedium, proved to be more effective than using DMSO alone. On assessment of the scaffolds’ mechanical properties after cryopreservation, microcracks were found that might have been formed because of ice expansion in the pores of the structure. Although the method of “open air” freezing (the name given by the authors) minimizes the crystallization of residual cryoprotectors, the destructive effect of the ice on the mechanical properties of the 3D structures could not be completely eliminated [132].

Cagol, N. et al. [37] studied the influence of cryopreservation on the viability and functionality of cells encapsulated in alginate scaffolds by comparing various cryoprotective agents. For the study, the authors used human MG63 osteosarcoma cells encapsulated in sodium alginate fibrous constructs and exposed them to various cryopreservative media containing dimethyl sulfoxide (DMSO), glycerol, and trehalose. Then the cell-loaded alginate fibers were slowly cooled to −80 °C and stored in liquid nitrogen. The viability of the cells encapsulated in the alginate scaffold after thawing varied from 71% to 85% depending on the composition of the cryoprotective medium. Cells cryopreserved under encapsulated conditions and then released from the hydrogel demonstrated greater metabolic activity and proliferative capacity compared to cells cryopreserved in suspension. After freezing in the presence of various cryoprotectors, in the short and medium term (up to 2 weeks after thawing) cryopreserved encapsulated cells showed a faster functional recovery than did cells frozen in suspension. The authors position the described cryofreezing protocol as a method to produce and store cell-loaded hydrogel structures that can serve as building blocks for the subsequent assembly of tissue structures in accordance with various biotechnological strategies [37].

Other 3D biomedical cell-based products used in regenerative medicine are spheroids. These lack an artificial extracellular matrix, their three-dimensional structure being maintained due to the initial aggregation of the cells and their subsequent production of a natural extracellular matrix. Jeong, Y.H. et al. [70] compared slow freezing and vitrification for both a one-layer stem cell culture and a 3D structure involving MSC spheroids. The study results showed the advantage of vitrification in the case of cell aggregates; moreover, greater spheroid size increased the viability of the MSCs. In the case of the one-layer culture, both freezing protocols showed comparable results.

When preparing this review, the authors came to the conclusion that, currently, there are many studies assessing the impact of cryopreservation on the viability, morphology, and functional activity of cells. However, studies related to three-dimensional, biomedical, cell-based products are rare and do not allow for extrapolation of the results to all 3D structures. The authors in their own research [133] have attempted to assess the possibility and conditions of cryopreservation required for a hybrid hydrogel scaffold that can ensure both the viability and proliferative activity of its cell-based component after long-term cryopreservation. The proposed protocol allows the frozen scaffold to maintain both high cell viability and proliferative activity for three months. The authors identified the phasing character of changes that occur with the scaffold and cells during storage for periods exceeding three months (6 months—a decrease in cell proliferative activity; 9 months—decreases in both viability and proliferative activity of the cells; 12 months—changes in the hydrogel structure and a reduction in cell viability and proliferative activity), which highlights the value of developing protocols for longer cryopreservation that can slow down or eliminate the observed changes over these periods.

The authors hereof studied the process of recovery after cryopreservation of cell cultures encapsulated in a scaffold. In this case, the scaffolds were cultivated with cells for 96 h after thawing, and they demonstrated a statistically significant increase in the total number of cells. It should be noted that the number of cells after cryopreservation and 96-h recovery was comparable to the number of cells in scaffolds without cryopreservation. It should also be noted that one of the conclusions made as a result of this work was the need to develop protocols for the assessment of the quality of such products before and after cryopreservation, to provide for comparable assessment of the viability and functional activity of the cells, as well as of the scaffold condition. This will allow for assessing the preservation of regenerative potential of the product after cryopreservation and provide opportunities for developing techniques to avoid the loss of therapeutic effect should the product be used later on. The protocol for the cryopreservation of scaffolds with encapsulated MSCs presented in the study could provide the basis for the development of new protocols for the storage of such tissue-engineered products. This can also support scaling up of their clinical use and accelerate their commercialization.

## 7. Conclusions

Summarizing the materials presented in the review, one can conclude that MSC cryopreservation technology, despite its widespread application in biobanking, is difficult to standardize, largely due to the variability of its different phases. Selection of a cryoprotector and a cryoprotective medium, as one of the first steps in cryopreservation, probably shows the greatest variation. Such variability is due to the ongoing search for new solutions both in relation to cryoprotectors and to optimization of the composition of the cryoprotective mixture. Here, the development of multicomponent media is becoming the most promising means of optimization, as—due to the combination of various substances—it provides for the creation of a non-toxic freezing medium with a high cryoprotective effect. It should be noted that optimization and standardization of the selection of a cryoprotective medium will, to a large extent, facilitate the standardization of the entire cryopreservation process, because variability of the subsequent phases will essentially depend on the chosen cryoprotector and its concentration. For example, incubation at physiological temperatures is required when using endocellular cryoprotectors, but this is not the case with exocellular protectors; at low CPA concentrations, a slow freezing protocol is implemented and samples can subsequently be stored at any temperature (−80 °C, −150 °C, or in liquid nitrogen), whereas at high CPA concentrations, only vitrification and storage in liquid nitrogen can be used.

In the case of cryopreservation of MSC suspensions, increased attention should be given to standardization of assessment of the cell culture quality. Here, comparability of the assessment criteria before and after cryopreservation is of particular importance. Comparison of similar indicators before and after freezing will provide an accurate understanding of the impact of the cryopreservation on the cell culture. Moreover, one should bear in mind that the cell culture needs to be restored or acclimatized after cryopreservation. Several studies have demonstrated that it is impossible to fully restore the therapeutic potential of cryopreserved stem cells without this acclimatization stage.

The cryopreservation of intact tissues or parts of tissues is of growing interest, which is also associated with the cell “niche” concept. However, although there are quite a few studies related to tissue cryopreservation, the most recent protocols for tissue cryopreservation are in many respects similar to the protocols for cell suspension cryopreservation, and require more detailed study, refinement, and optimization.

The “niche” concept has also contributed to development of the idea of the cryopreservation of cellular engineered structures; however, at present there are only a few studies on the cryopreservation of specific, complex, biomedical cell-based products, including both the carrier scaffold and the cell-based component. It should be taken into account that different complex cell-based products can vary significantly, which makes a single cryopreservation protocol inapplicable to all of them. In turn, the development of a separate protocol for each specific product greatly complicates standardization. Thus, the cryopreservation of complex biomedical cell-based products is a complicated and debatable issue. However, as the research results indicate, the idea itself is viable and requires further detailed study.

It should also be noted that another factor that complicates standardization of cryopreservation is the wide variation in the technical equipment available in laboratories. As each laboratory develops an in-house cryopreservation protocol, it is very common practice for this simply to comply with the laboratory’s own technical capabilities and available reagents. Thus, an integrated approach is required to bring the cryopreservation process to a certain standard.

However, it should be possible to consolidate general recommendations to optimize the cryopreservation process, making it less traumatic for cells. The authors hope that this review will be useful in terms of the systematization and selection of approaches to the cryopreservation of MSCs, MSC-containing tissues, and the biomedical cell-based products based on them.

## Figures and Tables

**Table 1:** Cryoprotectors.

Principle of Operation		
endocellular (penetrating)	exocellular (non-penetrating)	
**Class of compounds by molecular weight**		
low molecular weight compounds	high molecular compounds	
**Substances**		
dimethyl sulfoxide (DMSO), glycerol, ethylene glycol, propylene glycol 1,2-propanediol, methanol, dimethylacetamide	oligosaccharides	high molecular polymer compounds
	sucrose, trehalose	ficoll, albumin, polyvinylpyrrolidone, hydroxyethyl starch, polyethylene glycol, hexamethylene hydroxyethyl urea, oxyethylated glycerin

## Data Availability

Not applicable.
